# Activation of Neuropeptide Y Receptors Modulates Retinal Ganglion Cell Physiology and Exerts Neuroprotective Actions *In Vitro*

**DOI:** 10.1177/1759091415598292

**Published:** 2015-08-25

**Authors:** João Martins, Filipe Elvas, Dan Brudzewsky, Tânia Martins, Bogdan Kolomiets, Pedro Tralhão, Casper R. Gøtzsche, Cláudia Cavadas, Miguel Castelo-Branco, David P. D. Woldbye, Serge Picaud, Ana R. Santiago, António F. Ambrósio

**Affiliations:** 1 Institute for Biomedical Imaging and Life Sciences (IBILI), Faculty of Medicine, University of Coimbra, 3004-548 Coimbra, Portugal; 2 CNC.IBILI, University of Coimbra, 3004-548 Coimbra, Portugal; 3 AIBILI, 3000-548 Coimbra, Portugal; 4 Sorbonne Universités, UPMC Univ Paris 06, Institut de la Vision, UMR_S968, 75012 Paris, France; 5 Laboratory of Neural Plasticity, Department of Neuroscience and Pharmacology, University of Copenhagen, 2200 Copenhagen, Denmark; 6 Center for Neuroscience and Cell Biology, University of Coimbra, 3004-504 Coimbra, Portugal; 7 Faculty of Pharmacy, University of Coimbra, 3000-548 Coimbra, Portugal

**Keywords:** calcium imaging, electrophysiology, neuromodulation, neuropeptide y, retinal explants, retinal ganglion cells

## Abstract

Neuropeptide Y (NPY) is expressed in mammalian retina but the location and potential modulatory effects of NPY receptor activation remain largely unknown. Retinal ganglion cell (RGC) death is a hallmark of several retinal degenerative diseases, particularly glaucoma. Using purified RGCs and *ex vivo* rat retinal preparations, we have measured RGC intracellular free calcium concentration ([Ca^2+^]_i_) and RGC spiking activity, respectively. We found that NPY attenuated the increase in the [Ca^2+^]_i_ triggered by glutamate mainly via Y_1_ receptor activation. Moreover, (Leu^31^, Pro^34^)−NPY, a Y_1_/Y_5_ receptor agonist, increased the initial burst response of OFF-type RGCs, although no effect was observed on RGC spontaneous spiking activity. The Y_1_ receptor activation was also able to directly modulate RGC responses by attenuating the NMDA-induced increase in RGC spiking activity. These results suggest that Y_1_ receptor activation, at the level of inner or outer plexiform layers, leads to modulation of RGC receptive field properties. Using *in vitro* cultures of rat retinal explants exposed to NMDA, we found that NPY pretreatment prevented NMDA-induced cell death. However, in an animal model of retinal ischemia-reperfusion injury, pretreatment with NPY or (Leu^31^, Pro^34^)−NPY was not able to prevent apoptosis or rescue RGCs. In conclusion, we found modulatory effects of NPY application that for the first time were detected at the level of RGCs. However, further studies are needed to evaluate whether NPY neuroprotective actions detected in retinal explants can be translated into animal models of retinal degenerative diseases.

## Introduction

Neuropeptide Y (NPY) is widely distributed in the central and peripheral nervous systems (Silva et al., 2005). This peptide belongs to a family of highly conserved peptides which also includes pancreatic polypeptide (PP) and peptide YY (Michel et al., 1998). During the last three decades, NPY has been associated with a multitude of physiological functions such as energy homeostasis (Sousa-Ferreira et al., 2011; Chambers and Woods, 2012), stress response (Hirsch and Zukowska, 2012), circadian rhythm (Yannielli and Harrington, 2004), bone physiology (Lee and Herzog, 2009), neurogenesis (Malva et al., 2012), and immune cell regulation (Dimitrijevic and Stanojevic, 2013). NPY, peptide YY, and PP activate seven transmembrane G protein-coupled receptors (GPCRs) named NPY receptors. All these receptors bind to G_i_/G_0_ proteins, which results in inhibition of adenylyl cyclase. Other signaling pathways are also regulated, such as Ca^2+^ channels and G protein-coupled inwardly rectifying potassium channels (Sun and Miller, 1999), phospholipase C (Perney and Miller, 1989), and phosphoinositide 3-kinase (Rosmaninho-Salgado et al., 2012). In humans, only four NPY receptors were cloned and known to be functionally active, Y_1_, Y_2_, Y_4_, and Y_5_ (Babilon et al., 2013). These receptors have been implicated as potential neuroprotective therapeutic targets because they can prevent neuronal cell death induced by excitotoxic insults (Silva et al., 2003; Xapelli et al., 2007).

In the retina, NPY presence has been demonstrated in different species. However, the localization of the various NPY receptors in retinal cells has been scarcely investigated, and its function in retinal physiology remains largely unknown (Santos-Carvalho et al., 2014). Previous works from our laboratory have shown that NPY exerts neuroprotective action against different toxic insults. Particularly, in primary rat mixed retinal cell cultures, NPY pretreatment prevented increased cell death induced by both 3,4-methylenedioxy-*N*-methylamphetamine and glutamate (Alvaro et al., 2008b; Santos-Carvalho et al., 2013a). In an animal model of excitotoxicity-induced retinal injury, intravitreal administration of NPY inhibited both the increase in cell death and retinal ganglion cell (RGC) loss induced by glutamate (Santos-Carvalho et al., 2013a). In addition, we and others presented evidence suggesting a neuromodulatory role of NPY in the retina. For example, NPY application regulates neurotransmitter release in the rabbit and chicken retinas (Bruun and Ehinger, 1993). Also, NPY attenuates depolarization-induced increase in [Ca^2+^]_i_ in primary mixed retinal cell cultures (Alvaro et al., 2009). Moreover, NPY decreases depolarization-dependent Ca^2+^ influx into bipolar cells via activation of Y_2_ receptors (D’Angelo and Brecha, 2004), and in retinas with selective ablation of NPY-expressing amacrine cells, an alteration in the receptive field properties of RGCs was reported, although a direct effect of NPY on RGCs was not demonstrated (Sinclair et al., 2004). These results suggest that NPY-induced modulation of visual circuitry might result in changes of RGC spiking activity. Therefore, in this study, we investigated the NPY modulatory effects at RGC level using a purified rat RGC culture and an *ex vivo* rat retinal preparation. In addition, since RGCs are lost in retinal degenerative diseases such as glaucoma, we also evaluated the neuroprotective potential of NPY against excitotoxic or ischemia-reperfusion (I-R) injuries.

## Material and Methods

### Animals

Wistar rats, 8 to 10 weeks old, were obtained from Charles River, France. Long Evans rats, 8 to 10 weeks old, were obtained from Charles River for RGC purification experiments and from Janvier Labs, Le Genest Saint Isle, France, for multielectrode array (MEA) experiments. Animals were provided with standard rodent diet and water *ad libitum* and kept on a 12 h light/12 h dark cycle. All procedures involving the animals were in agreement with the guidelines on the ethical use of animals from the European Community Council Directive 2010/63/EU.

### Drugs

NPY and NPY receptor agonists: (Leu^31^, Pro^34^)−NPY, NPY_13–36_, and (Gly^1^, Ser^3,22^, Gln^4,34^, Thr^6^, Arg^19^, Tyr^21^, Ala^23,31^, Aib^32^)-PP ((Gly^1^,…Aib^32^)-PP) were all obtained from Bachem, Switzerland. NPY receptor antagonists: BIBP 3226, BIBO 3304, BIIE 0246, and L-152,804 were obtained from Tocris Bioscience, UK. The other used reagents were obtained from Sigma-Aldrich, USA, unless stated otherwise.

### RGC Purification

Purified RGCs were obtained from the retinas of either 3 to 4 days old pups or 8 to 10 weeks old Wistar or Long Evans rats by a sequential immunopanning procedure yielding around 99% purity, as previously described (Barres et al., 1988), with some modifications, as follows. Rats were killed by decapitation or cervical dislocation, the eyes enucleated, and the retinas digested for 30 min at 37℃ in 16.5 U/mL papain (Worthington Biochemical, USA), 1.65 mM L-cysteine, and 124 U/mL deoxyribonuclease I (DNase I). The cell suspension was mechanically dissociated in 1.5 mg/mL ovomucoid (Roche, Switzerland), 1.5 mg/mL bovine serum albumin (BSA), and 124 U/mL DNase I in EBSS. The cell suspension was further triturated in 1.5 mg/mL ovomucoid, 1.5 mg/mL BSA, 124 U/mL DNase I, and 1:125 (v:v) rabbit anti-rat macrophage antiserum (Accurate Chemical, USA). After centrifugation for 11 min at 190 g at room temperature (RT), cells were resuspended in 10 mg/mL ovomucoid and 10 mg/mL BSA, and then centrifuged again for 10 min, at 190 g, at RT. Cells were resuspended in 0.2 mg/mL BSA and 5 µg/mL insulin. Cell suspension was plated in a goat anti-rabbit IgG (Rockland Immunochemicals, USA) coated dish. After 30 min at RT, nonadherent cells were removed to a second dish. After 30 min at RT, nonadherent cells were removed to a dish coated with goat anti-mouse IgM (Rockland Immunochemicals) and mouse anti-rat Thy1.1 hybridoma supernatant of T11D7e cell line (TIB-103, ATCC, USA). After 30 min, the nonadherent cells were removed, and RGCs were detached with a 0.125% trypsin solution. Trypsinization was stopped with 30% FBS (Gibco, Life Technologies, USA) in Neurobasal-A (Gibco). After final centrifugation for 10 min at 190 g, at RT, RGCs were resuspended. For cell culturing, RGCs were resuspended in Neurobasal-A medium containing 1 × B27 supplement (Gibco), 5 µg/mL insulin, 1 mM sodium pyruvate (Gibco), 1 × Sato/Bottenstein supplement (which includes 100 µg/mL transferrin, 100 µg/mL BSA, 16 µg/mL putrescine, 62 ng/mL progesterone, and 40 ng/mL sodium selenite), 40 ng/mL triiodo-L-thyronine, 2 mM L-glutamine, 5 mg/mL N-acetylcysteine, 100 µM inosine, 20 ng/mL ciliary neurotrophic factor and 25 ng/mL brain-derived neurotrophic factor (both from Peprotech, USA), 5 µM forskolin, 10 ng/mL basic fibroblast growth factor (Gibco), and 50 µg/mL gentamicin (Gibco) and were plated at a density of 460 cells/mm^2^ on 12 mm glass coverslips coated with 10 µg/mL poly-D-lysine and 10 µg/mL laminin. Cells were cultured for 16 to 48 h at 37℃ in a humidified environment of 5% CO_2_. For RNA extraction, the cell pellet was lysed in TRIzol reagent, as further described below. Regarding reverse-transcription polymerase chain reaction (RT-PCR), we extracted RNA immediately after the isolation of RGCs, and so we did not analyze mRNA expression from cultured purified RGCs. However, in RGC cultures, we did evaluate their purity by immunocytochemistry. The percentage of Brn3a^+^/DAPI^+^ cells varied between 70 and 98%. We also analyzed the mRNA expression of GFAP (marker of astrocytes and Muller cells) and CD11b (marker of microglia/macrophages) in some RGC isolations, and we did not find expression of these markers (data not shown).

### RT-PCR

Total RNA was isolated from RGCs using TRIzol reagent (Ambion, Life Technologies, USA). Subsequently, cDNA first strand synthesis was performed from 2 µg DNase-treated RNA using random primers and SuperScript II Reverse Transcriptase (Invitrogen, Life Technologies, USA). The resulting cDNA (0.5 µL) was used for amplification of respective targets with AmpliTaq Gold DNA polymerase (Applied Biosystems, Life Technologies, USA), 200 nM of primer and 2 mM MgCl_2_, in a Veriti thermal cycler (Applied Biosystems). Reactions were performed as follows: denaturation for 3 min at 95℃; followed by 40 cycles each consisting of 30 s at 95℃, 30 s at annealing temperature, and 30 s at 72℃; and final extension at 72℃ for 5 min). The following primers were used (indicated as forward, reverse, annealing temperature, and product size): NPY, AGAGATCCAGCCCTGAGACA, TTTCATTTCCCATCACCACA, 57℃, 110 bp; Y_1_, ACGTTCGCTTGAAAAGGAGA, CATGACGTTGATTCGTTTGG, 57℃, 89 bp; Y_2_, CAGTTTTGTGCCATTTGGTG, AGGAAGCTGATTTGCTTGGA, 60℃, 142 bp; Y_4_, ATCTCATGGCCTCCCTTTCT, TCTCAACGCTGTAGGTGGTG, 57℃, 141 bp; Y_5_, ATACAGCTGCTGCTCGGAAT, GATTGCCCATAAAGCCAAGA, 57℃, 126 bp. Reaction specificity was confirmed by running nontemplate control for each primer set. Equal volumes of PCR products were separated on a 1.5% agarose gel. The gel images were digitally acquired in a Gel/ChemiDoc (Bio-Rad Laboratories, USA), and the level of gene transcription was evaluated and categorized as detected or not detected.

### Immunofluorescence Labeling

For immunocytochemistry in purified RGC cultures, cells were fixed with 4% paraformaldehyde (PFA) for 20 min at RT and permeabilized with 1% Triton X-100 for 5 min. Unspecific binding was prevented with 3% BSA and 0.2% Tween 20 blocking solution for 60 min at RT. Cells were incubated with primary antibodies diluted in blocking solution for 90 min at RT as follows: rabbit anti-NPY (1:1000, Sigma-Aldrich), sheep anti-Y_1_ (1:500, AbD Serotec, Bio-Rad Laboratories, USA), rabbit anti-Y_2_ (1:500, Alomone Labs, Israel), rabbit anti-Y_4_ (1:25, Alomone Labs), rabbit anti-Y_5_ (1:250, Alomone Labs), or mouse anti-Brn3a (1:25, Merck Millipore, USA). Cells were incubated with the secondary antibodies diluted in blocking solution for 60 min at RT as follows: Alexa-Fluor 568 goat anti-mouse IgG (1:200), Alexa-Fluor 488 goat anti-rabbit IgG (1:200), or Alexa-Fluor 488 donkey anti-sheep IgG (1:200, all from Molecular Probes, Life Technologies, USA). The nuclei were stained with DAPI (2.5 µg/mL, Molecular Probes). Images were acquired in a laser scanning confocal microscope LSM 710 (Zeiss, Germany) using a 40 × numerical aperture (NA) = 1.3 objective lens, magnification 400×.

For immunohistochemistry in retinal sections, adult Wistar rats were transcardially perfused with 250 mL PBS followed by 250 mL 4% PFA at RT, under deep anesthesia induced by 90 mg/kg ketamine (Imalgene™) and 10 mg/kg xylazine (Rompun™). Then, the eyes were enucleated and fixed with PFA for 60 min. The cornea and lens were removed, and the eye cup was further fixed for 60 min in 4% PFA. Eye cups were transferred sequentially to 15% and 30% (w/v) sucrose for at least 120 min each. The eye cup was embedded in a mixture 1:1 of 30% sucrose and cryomatrix embedding resin (Thermo Fisher Scientific, USA), and stored at −80℃. Retinal sections, 10 µm thickness, were obtained in a cryostat and collected on SuperFrost Plus glass slides (Menzel-Glaser, Thermo Fisher Scientific) and stored at −20℃. Retinal sections were air dried for at least 45 min at RT and were fixed with acetone for 10 min at −20℃ and permeabilized in 0.25% Triton X-100 for 30 min at RT. In the case of Y_1_, Y_2_, Y_4_, and Y_5_ receptors, the fixation and permeabilization were replaced by an antigen retrieval step with 10 mM sodium citrate, pH 6.0, for 30 min at 95℃. The sections were permeabilized in 0.25% Triton X-100 for 30 min at RT, and blocked in 1% BSA and 10% goat serum for 30 min at RT. Sections were incubated overnight at 4℃ with primary antibodies diluted in 1% BSA in PBS: rabbit anti-NPY (1:10000), rabbit anti-Y_1_ (1:100) (Immunostar, USA), rabbit anti-Y_2_ (1:2000), rabbit anti-Y_4_ (1:200), rabbit anti-Y_5_ (1:2000), mouse anti-Brn3a (1:500), or mouse anti-vimentin (1:500; Lab Vision, USA). Sections were incubated for 60 min at RT with the corresponding secondary antibodies diluted in 1% BSA in PBS: Alexa-Fluor 488 goat anti-mouse IgG (1:500) or Alexa-Fluor 568 goat anti-rabbit IgG (1:500). The nuclei were stained with DAPI (2.5 µg/mL). The specificity of anti-NPY and anti-NPY receptor antibodies has been confirmed by the corresponding manufacturer. Images were acquired in a laser scanning confocal microscope LSM 710 using a 20 × NA = 0.8 objective lens, magnification 200×.

### NPY-Stimulated [^35^S]GTPγS Functional Binding

Eyes of 8 to 10 weeks old male Wistar rats were enucleated and frozen in dry ice. Retinal sections (18 µm thickness) were obtained in a cryostat, collected onto SuperFrost Plus glass slides and stored at −80℃ until further processing. Sections were air dried for 30 min at RT and then rehydrated in assay buffer A (50 mM Tris-HCl, 3 mM MgCl_2_, 0.2 mM EGTA, 100 mM NaCl, pH 7.4) for 10 min at RT. Sections were preincubated in assay buffer B (assay buffer A + 0.2 mM dithiothreitol, 1 µM 1,3-dipropyl-8-cyclopentylxanthine (DPCPX), 0.5% w/v BSA, and 2 mM GDP) for 15 min at RT to shift all G proteins into the inactive state. Subsequently, incubation of retinal slices was performed in assay buffer B + 50 pM [^35^S] guanosine 5’-[γ-thio]triphosphate (GTPγS; 1250 Ci/mmol; PerkinElmer, USA) for 60 min at RT with 1 to 10 µM NPY. In each experiment, basal binding was determined by incubation without NPY receptor ligands but with assay buffer B + 50 pM [^35^S]GTPγS (1250 Ci/mmol). Specificity was confirmed by adding a combination of the NPY receptor antagonists: 10 µM BIBO 3304 for Y_1_; 10 µM BIIE 0246 for Y_2_; 100 µM L-152,804 for Y_5_. For antagonistic studies, NPY receptor antagonists were also added to the preincubation buffer B. Incubation was terminated by two washes of 5 min each in ice cold 50 mM Tris-HCl buffer, pH 7.4, followed by a final wash in deionized water. Sections were air dried at RT and exposed to Kodak BioMax MR films (Carestream Health, Rochester, USA) together with ^14^C-microscales (Amersham, GE Healthcare, Little Chalfont, UK) for 5 days at −20℃. The films were developed in Kodak GBX developer, and the optical densities of retinal slices measured using ImageJ software (http://imagej.nih.gov/ij). The values obtained were converted to estimated nCi/g tissue using ^14^C-microscales.

### Fura-2 Intracellular Ca^2+^ Imaging in Purified RGCs

Purified RGCs cultured for 1 or 2 DIVs were used to assess the changes in [Ca^2+^]_i_ using the Ca^2+^ dye Fura-2-AM, with a fluorescence microscope Axiovert 200 (Zeiss) coupled to a perfusion system. RGCs were loaded with 5 µM Fura-2-AM in the presence of 0.02% Pluronic F-127 (both Molecular Probes, Life Technologies, USA) for 45 min at 37℃, in HBSS Mg^2+^-free solution (in mM: 138 NaCl, 5.3 KCl, 0.34 Na_2_HPO_4_, 0.44 KH_2_PO_4_, 2.6 CaCl_2_, 5.6 glucose, 15 HEPES, 4.17 NaHCO_3_, pH 7.4) supplemented with 0.1% fatty acid-free BSA. Under continuous perfusion (2.9 ± 0.1 mL/min) with HBSS Mg^2+^-free solution, RGCs were exposed to glutamate for 30 s, and all glutamate stimuli included 10 µM glycine, a coagonist of *N*-methyl-D-aspartic acid (NMDA) glutamate receptor, as previously described (Hartwick et al., 2004). RGCs were alternately excited at 340 and 380 nm, and a ratio of fluorescence intensity (340 nm/380 nm) was calculated for each individual cell by Metafluor software (Molecular Devices, Sunnyvale, USA). Fura-2 ratios (R) were converted to [Ca^2+^]_i_ in separate calibration experiments using the following formula: [Ca^2+^]_i_ = [K_d_(F_0_/F_S_)][R – R_min_)/(R_max_ – R)], with K_d_ for Fura-2 of 224 nM, and where F_0_/F_S_ is the ratio of fluorescence intensity at 380 nm excitation in Ca^2+^-free solution over the intensity in solution with saturated Ca^2+^ levels (Hartwick et al., 2004). The minimum value for the Fura-2 ratio (R_min_) was obtained using Ca^2+^-free HBSS and 1 µM ionomycin, after which the cells were perfused with Mg^2+^-free HBSS and 1 µM ionomycin in order to calculate the maximum value for Fura-2 ratio (R_max_).

### Ex Vivo MEA Recordings

Long Evans rats (8 weeks old) were dark-adapted for at least 12 h and killed by CO_2_ inhalation and quick cervical dislocation under dim red light. Eyes were enucleated and placed in oxygenated Ames’ medium at RT. Square pieces of retina (1–2 mm^2^) were placed into the recording chamber, with the ganglion cell layer (GCL) facing the MEA60 biochip electrode array. The electrode array was composed of 60 titanium nitride electrodes, 10 µm diameter each, disposed in a 8 × 8 layout with 100 µm interelectrode spacing (Multi Channel Systems—MCS, GmbH, Germany). Retinas were held in the center of the electrode array using a piece of polycarbonate membrane covered by a U-shaped platinum ring with a nylon mesh. During recording sessions, retinas were continuously perfused with Ames’ medium equilibrated with 95% O_2_ and 5% CO_2_, pH 7.4, at a flow rate of 1.3 mL/min. Retinas were maintained at 34 to 37℃ through a TC01 heating pad (MCS) of the recording system. In order to obtain stable recordings, each session started 60 min after placing the retina in MEA recording chamber. MEA recordings were conducted using MEA60 setup (MCS). The analog extracellular neuronal signals from 60 channels were AC amplified (×1000–1200), band-pass filtered (200–3000 Hz), sampled at 20 to 30 kHz, and saved in PC-compatible computer for subsequent off-line analysis. RGC spiking activity was monitored during recording sessions using MC_Rack software (MCS). Light-induced responses were evaluated under dark conditions. To elicit light responses in the RGCs, white light stimulation blocks produced from light-emitting diodes driven by a stimulus generator STG-1008 (MCS) were applied. The light-emitting diodes were positioned 5 mm below the transparent MEA chamber and used to generate full-field stimuli in the photopic range (5.0 cd/m^2^). Stimuli consisted of 10 consecutive stimulus blocks with 5 s light followed by 10 s dark each. The recordings were subsequently subjected to off-line spike sorting and analysis using Spike2 (Cambridge Electronic Design, UK). Waveforms were isolated using a combination of template matching and cluster cutting. The spontaneous activity was calculated for each RGC as spiking rate (Hz). To detect changes in spontaneous activity induced by light, the raster and peri-stimulus time histograms were generated from 10 stimulus blocks using 50 ms bin widths. The onset of ON- and OFF-type RGC responses were defined as an increase in spike number higher than two standard deviations than the prestimulus frequency over at least three consecutive bins. The initial burst responses to both light onset (ON-type RGCs) and light offset (OFF-type RGCs) were quantified over a 50 ms bin width. Latency was defined as the time delay between light onset or offset (ON- or OFF-type RGCs, respectively) and the RGC light response as defined above, when aligned in raster plots for nine consecutive light stimuli.

### Culture of Retinal Explants and Cell Death Evaluation

Wistar rats (8–10 weeks old) were killed by cervical dislocation. Retinas were flat-mounted onto 30 mm diameter culture plate hydrophilic polytetrafluoroethylene inserts with a 0.4 µm pore size (Millicell, Merck Millipore), with the GCL side facing upward. The retinal explants were cultured in Neurobasal-A media containing 1 × B27 supplement, 2 mM L-glutamine, and 50 µg/mL gentamicin, and maintained for 4 DIVs in a humidified environment of 5% CO_2_ at 37℃. To induce cell death, cultured retinal explants were exposed to 300 µM NMDA at DIV2.

For TdT-mediated dUTP nick-end labeling (TUNEL) assay, the manufacturer’s instructions were followed (Promega, USA). Briefly, retinal explants were fixed in 4% PFA for 15 min at RT and permeabilized with 20 µg/mL proteinase K for 15 min at RT followed by further fixation in 4% PFA for 5 min. Retinal explants were incubated with the recombinant TdT enzyme and nucleotide mix containing dUTP conjugated to fluorescein at 37℃ for 60 min. The reaction was stopped by incubating the retinal explants in saline-citrate buffer for 15 min at RT. The nuclei were stained with DAPI (2.5 µg/mL). At least 12 images of GCL per retinal explant (three images per quadrant) were acquired in a laser scanning confocal microscope LSM 710 using a 40 x NA = 1.3 objective lens, magnification 400×. TUNEL-positive cells localized in GCL were quantified, since this layer is highly affected in diseases like glaucoma, and the values were then normalized to the value obtained in nonNMDA treated retinal explant (control), in each independent experiment.

For propidium iodide (PI) incorporation assay, cultured retinal explants were incubated with 2 µM PI for 180 min at DIV2 and at DIV4. Since the acquisition of images was performed with live retinal explants using fluorescence microscopy (DM IRE2, Leica, Germany) with a 10 × NA = 0.22 objective lens, magnification 100×, we quantified PI-positive cells across all retinal layers. Images comprising the four quadrants of retinal explant were acquired, and the PI-positive cells were counted at DIV2 (before NMDA treatment and at DIV4). The extent of cell death was expressed as the ratio between PI-positive cells at DIV4 and DIV2.

### Intravitreal Injections and Retinal I-R Injury

Wistar rats (8–10 weeks old) were anesthetized by 2.5% isoflurane inhalation using a gas-anesthetizing system (VetEquip, USA). Then, 4 mg/mL oxybuprocaine (Laboratórios Edol, Portugal) anesthetic was applied topically to the eyes and the pupils dilated with 10 mg/mL tropicamide (Laboratórios Edol). Intravitreal injection of 5 µL containing 10 µg (2.34 nmol) NPY, 10 µg (2.36 nmol; Leu^31^, Pro^34^)−NPY, or sterile saline solution was performed using a 10 µL Hamilton syringe (Hamilton, USA) with a 30-gauge needle, in both eyes, 120 min before the induction of retinal I-R injury. Fusidic acid (10 mg/g, Leo Pharmaceutical, Denmark) ointment was applied in the conjunctival sac after the intravitreal injections. Retinal I-R injury was induced in one eye by elevating the intraocular pressure (IOP) to 80 mmHg for 60 min. IOP was measured with a tonometer (Tonolab, Icare, Finland). The anterior chamber of one eye was cannulated with a 30-gauge needled connected to a reservoir infusing sterile saline solution. The contralateral eye was taken as the control eye. The IOP was raised by elevating the reservoir to a height of 1.8 m. Retinal ischemia was confirmed by whitening of the iris and loss of the red reflex. In order to avoid corneal opacity, 2% methocel™ (Dávi II, Portugal) was applied to both eyes. Reperfusion was confirmed by returning of red color to the iris and the red reflex to the eye fundus. Hypothermia was avoided using warmed blankets covering the animals during all the procedure. After removing the needle from the anterior chamber, the animals were observed until full recovery which occurred within 5 min. Animals were killed after 24 h of reperfusion. There were no animals excluded from the study.

### Electroretinogram Recordings

Electroretinograms (ERGs) were recorded before the onset of I-R injury (baseline) and after 24 h of reperfusion. After dark adaptation for at least 12 h, the animals were anesthetized with 90 mg/kg ketamine and 10 mg/kg xylazine (i.p). Then, 4 mg/mL oxybuprocaine anesthetic was applied topically to the eyes and the pupils dilated with 10 mg/mL tropicamide under dim red light illumination. The body temperature was maintained with a heating pad set to 37℃. Using a Ganzfeld stimulator, white light flashes (0.0095–9.49 cd-s/m^2^) were applied under scotopic and photopic conditions (in the latter case, after 16 min of light adaptation to a white background, 25 cd/m^2^). ERGs were recorded with a corneal gold wire electrode, a reference electrode at the head and a ground electrode in the tail of the animal. A band width of 1 to 300 Hz and sampling rate of 3.4 kHz were used for acquisition (Roland Consult GmbH, Germany). The scotopic a-wave and b-wave, and photopic b-wave, were evaluated. Off-line digital filter was applied on b-wave (high frequency cutoff of 50 Hz) with the RETIport software (Roland Consult GmbH).

### Brn3a Labeling and TUNEL Assay in Retinal Sections

The animals subjected to I-R injury were used for Brn3a labeling by immunohistochemistry with antibody mouse anti-Brn3a (1:500, Merck Millipore, USA), using the same protocol as for retinal sections. After this procedure, a TUNEL assay was performed following manufacturer’s instructions (Promega). Briefly, sections were permeabilized with 20 µg/mL proteinase K for 10 min at RT and incubated with the recombinant TdT enzyme and nucleotide mix containing dUTP conjugated to fluorescein at 37℃ for 60 min. The reaction was stopped by immersing the slides in saline-citrate buffer for 15 min at RT. The nuclei were stained with DAPI (2.5 µg/mL). Images of retinal sections were acquired in a fluorescence microscope. The Brn3a- and TUNEL-positive cells were counted, and results were expressed per mm of GCL length. Images were acquired using a fluorescence microscope (DM IRE2, Leica) with a 20 × NA = 0.3 objective lens, magnification 200×.

### Statistical Analysis

Statistical analysis was performed with Prism 5 (GraphPad, USA) using one-way analysis of variance followed by Bonferroni’s test. When distribution normality was not achieved, one-way Kruskal-Wallis test was used followed by Dunn’s test, as indicated in figure legends. *P* values less than 0.05 were taken as significant. All values are presented as mean ± SEM.

## Results

### Expression of NPY and NPY Receptors in RGCs

**Figure 1.**
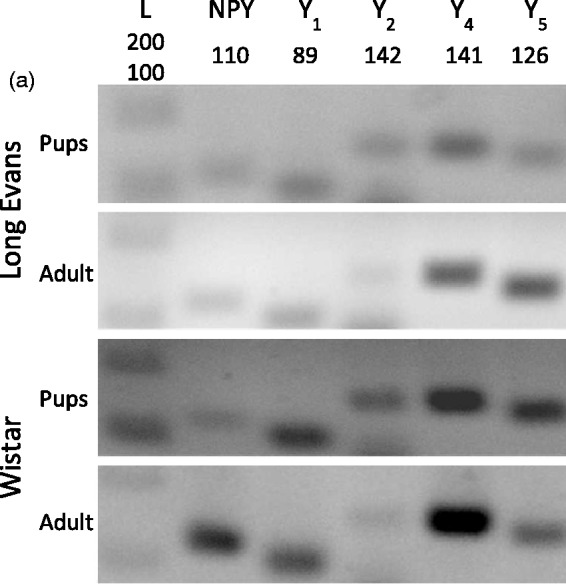

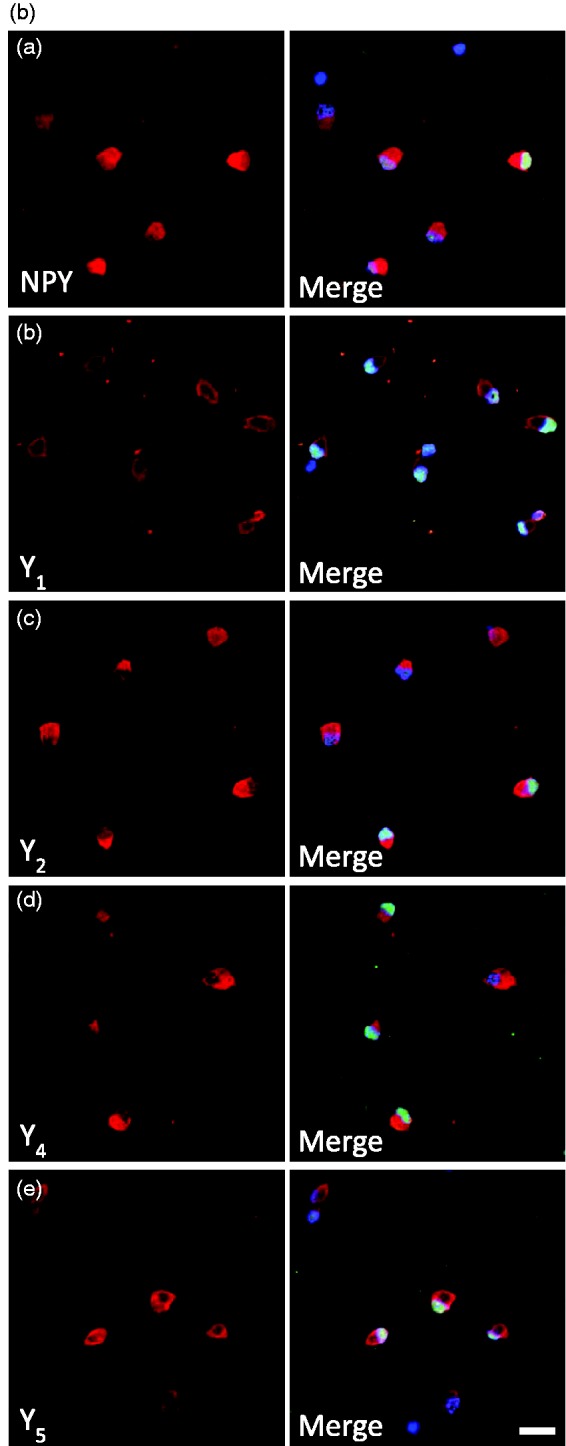

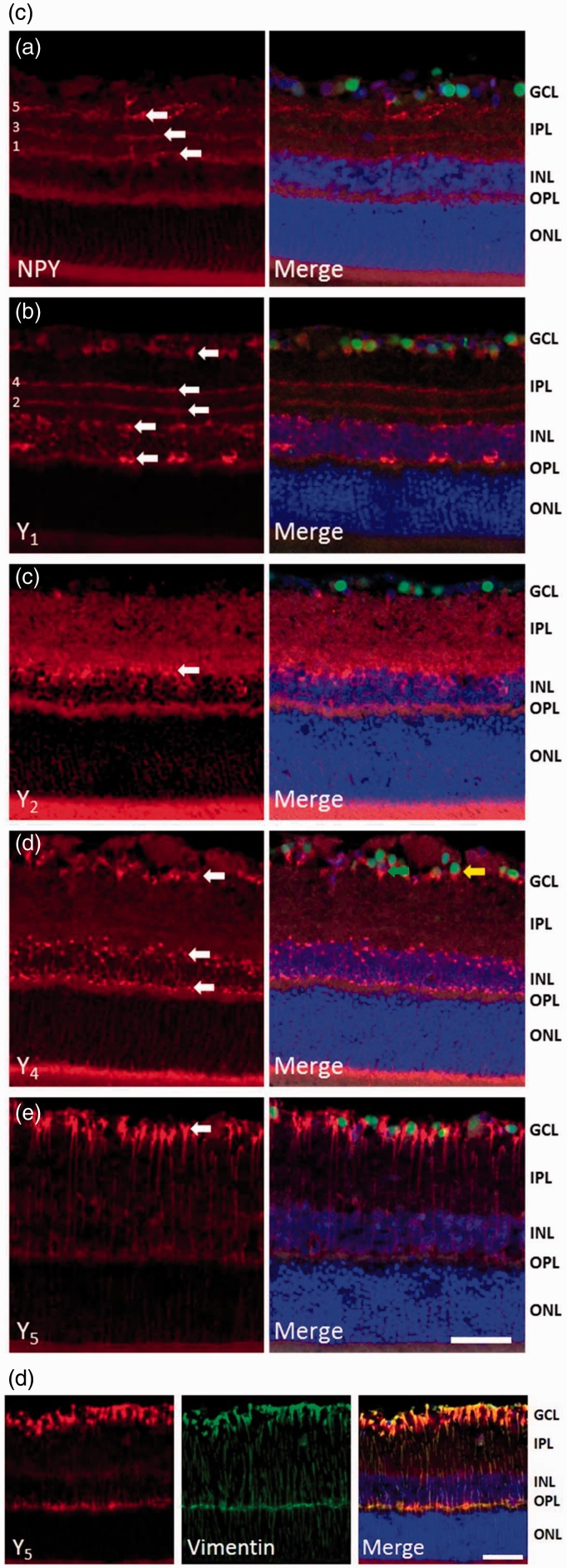
Expression of NPY and NPY receptors in RGCs. (a) Detection of NPY and NPY receptors (Y_1_, Y_2_, Y_4_, and Y_5_) mRNA expression by RT-PCR in purified RGCs. RGCs were isolated from the retinas of either pups or young adult Long Evans and Wistar rats. DNA ladder. (b) NPY and NPY receptor immunoreactivity (ir) in a purified RGC culture obtained from Wistar pups (a–e, red). RGCs were stained with antiBrn3a (RGC marker, green) and nuclei with DAPI (blue). Scale bar: 20 µm. (c) NPY-ir and NPY receptor-ir in retinal slices obtained from young adult Wistar rats (a–e, red). RGCs were stained with the RGC marker Brn3a (green) and nuclei with DAPI (blue). NPY-ir was mainly detected in strata 1, 3, and 5 of IPL (arrows; a). Y_1_-ir was detected in distal and proximal INL, strata 2 and 4 of IPL, and RGCs (arrows; b). Y_2_-ir was detected in INL (c). Y_4_-ir was detected in INL and GCL (d). and Y_5_-ir was detected in Müller cells (e). (d) Y_5_-ir (red) is colocalized with Müller cells (green) in retinal slices of young adult Wistar rats. Müller cells were identified by vimentin-ir (green). Nuclei were stained with DAPI (blue). GCL = ganglion cell layer; IPL = inner plexiform layer; INL = inner nuclear layer; OPL = outer plexiform layer; ONL = outer nuclear layer. Scale bar: 50 µm.

The presence of NPY and NPY receptors (Y_1_, Y_2_, Y_4_, and Y_5_) was assessed in purified rat RGCs. Total RNA was extracted immediately after purification. To assess the presence of mRNA encoding for NPY and NPY receptors (Y_1_, Y_2_, Y_4_, and Y_5_) by RT-PCR, we used cDNA from RGCs isolated from the retinas of P3 to P4 pups and 8 weeks old adult rats of two different strains: Wistar and Long Evans (Figure 1(a)). We found that mRNA for both NPY and NPY receptors (Y_1_, Y_2_, Y_4_, and Y_5_) was detected in purified RGCs from both rat strains and at both ages.

To assess the NPY and NPY receptor expressions at protein level, we also evaluated their immunoreactivity (ir) in purified RGCs from P3 to P4 Wistar rats cultured overnight. We found NPY-ir in RGCs with colocalization with the RGC marker Brn3a (Figure 1(ba)). Similar to NPY-ir, immunoreactivity for Y_1_, Y_2_, Y_4_, and Y_5_ receptors was also detected in RGCs (Figure 1(bb–e)). In addition, we assessed the localization of NPY and NPY receptors in retinal sections of adult Wistar rats. NPY-ir was detected in ramified dendrites in strata 1, 3, and 5 of inner plexiform layer (IPL; Figure 1(ca)), and cell bodies of GCL and inner nuclear layer (INL). Similar observations were reported previously, identifying these cells as amacrine cells (Oh et al., 2002). Y_1_ receptor-ir was detected in cell bodies in the distal and proximal INL (Figure 1(cb)), arrows). A previous study has identified these cells as horizontal and amacrine cells (D’Angelo et al., 2002). In accordance, we also detected Y_1_-ir processes ramifying in strata 2 and 4 of IPL. In addition, we found that some Brn3a-ir RGCs colocalized with Y_1_. We also detected, for the first time, Y_2_ receptor-ir in stratum 1 of IPL and in cell bodies in proximal INL (Figure 1(cc)). Y_4_ receptor-ir was localized in cell bodies of GCL, and proximal and distal INL (Figure 1(cd)). The cell bodies in GCL that were immunoreactive for Y_4_ receptor were both RGC (colocalized with Brn3a, yellow arrow) and nonBrn3a-positive cells, likely displaced amacrine cells (green arrow). Concerning the Y_5_ receptor, immunoreactivity was detected in Müller cells (Figure 1(ce)). Müller cell identity was confirmed by colocalization with vimentin (Figure 1(d)). This is the first time the localization of Y_4_ and Y_5_ receptor was assessed in rat retinal sections. The lack of clear immunoreactivity for NPY, and for Y_2_ and Y_5_ receptors in RGCs in retinal sections of adult rat, while it could be found in cultured RGCs from P3 to P4 rats, may be explained by decreased expression in adulthood.

### NPY Stimulates Functional Binding in Retinal Slices

**Figure 2.**
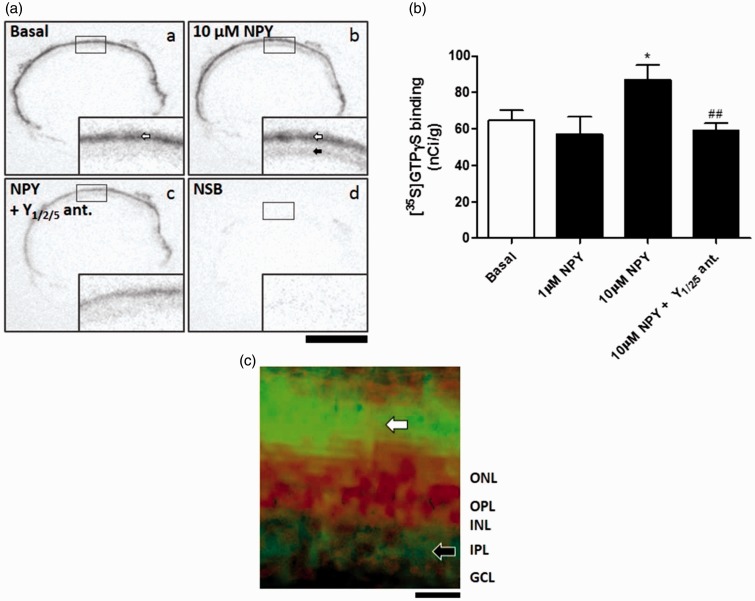
NPY stimulates [^35^S]GTPγS functional binding in retinal sections. (a) Examples of retinal sections from autoradiography films using [^35^S]GTPγS-binding assay. (b) Quantification of [^35^S]GTPγS binding in inner retinal layers. Incubation with 10 µM NPY increased [^35^S]GTPγS binding in inner retinal layers (ab, black arrow) compared with basal binding (aa). (c) [^35^S]GTPγS binding signal (green) merged with hematoxylin-eosin staining (red). The green represents the [^35^S]GTPγS binding signal as in Figure 2ab, where the black was converted to green in order to be better visualized in the merged picture with hematoxylin-eosin staining which is in red. Note the increased signal in IPL (black arrow). An intense signal was found in the photoreceptor layer (white arrows) both under basal and NPY-stimulated binding conditions that may represent the high amount of G proteins in photoreceptor outer segments, mainly transducin. The blockade of Y_1_, Y_2_, and Y_5_ receptors prevented the increased [^35^S]GTPγS binding induced by NPY (ac). NSB refers to nonspecific binding, a competitive control with nonradioactive GTPγS (ad). Antagonists used: Y_1_, BIBO 3304 (10 µM); Y_2_, BIIE 0246 (10 µM); Y_5_, L-152,804 (100 µM). Bar: 2 mm. Data are presented as mean ± SEM of *n* = 2 to 10 independent experiments. *p < .05, compared with basal; ^##^*p* < .01, compared with 10 µM NPY. One-way analysis of variance followed by Bonferroni’s test.

We performed [^35^S]GTPγS-binding assay (Christensen et al., 2006) to assess the functional activity of NPY receptors in retinal slices from adult Wistar rats (Figure 2). [^35^S]GTPγS-binding assay is able to evaluate the activation of GPCRs taking advantage of a radiolabeled nonhydrolyzable GTP analog—[^35^S]GTPγS. Upon activation of the GPCR by a receptor agonist, Gα binds [^35^S]GTPγS allowing the measurement of the amount of radiolabeled GTP bound to the cell membrane using an autoradiography film. After incubation with 10 µM NPY for 60 min (*n* = 10 animals), we detected an increase in [^35^S]GTPγS binding in inner retinal layers comparing to basal conditions (*n* = 10)—nonstimulated retinal slices; Figure 2(a) and (b). The small size of the rat retinal tissue and the resolution of autoradiography films made it difficult to identify the exact part of the inner retinal layers responsible for the increased NPY-stimulated [^35^S]GTPγS functional binding. However, when comparing the hematoxylin-eosin staining of native retinal slices with autoradiography film pictures of the same retinal slices, we found that the retinal layers displaying increased [^35^S]GTPγS binding upon NPY stimulation corresponded to the GCL and IPL (Figure 2(c)). It is also of note that an intense binding signal corresponding to the photoreceptor layer was detected both under basal and NPY-stimulated conditions (Figure 2(aa) and (ab), white arrows). This likely represents the high amount of G proteins in the photoreceptor outer segments, mainly transducin (Arshavsky et al., 2002). Moreover, NPY did not induce a statistically significant increase in binding signal in the photoreceptor layer (data not shown). Since 1 µM NPY (*n* = 2, not used for statistical tests) was not sufficient to increase [^35^S]GTPγS binding in retinal sections, we speculated that low levels of NPY receptors were functionally active in frozen retinal sections, thus requiring increased concentrations of NPY. A cocktail of NPY receptor antagonists (10 µM BIBO 3304—Y_1_ receptor antagonist; 10 µM BIIE 0246 for Y_2_; 100 µM L-152,804 for Y_5_) was used to evaluate the selectivity of NPY-stimulated [^35^S]GTPγS binding (Figure 2(ac) and (b)). The blockade of Y_1_, Y_2_, and Y_5_ receptors (*n* = 8) prevented the increased [^35^S]GTPγS binding induced by NPY, confirming the selectivity of the signal corresponding to the GCL and IPL. To assess the nonspecific binding, a competitive control with nonradioactive GTPγS was used, which exhibit no clear binding signal in the autoradiography film (Figure 2(ad)).

### NPY Attenuates Glutamate-Induced [Ca^2+^]i Increase in Purified RGCs

**Figure 3.**
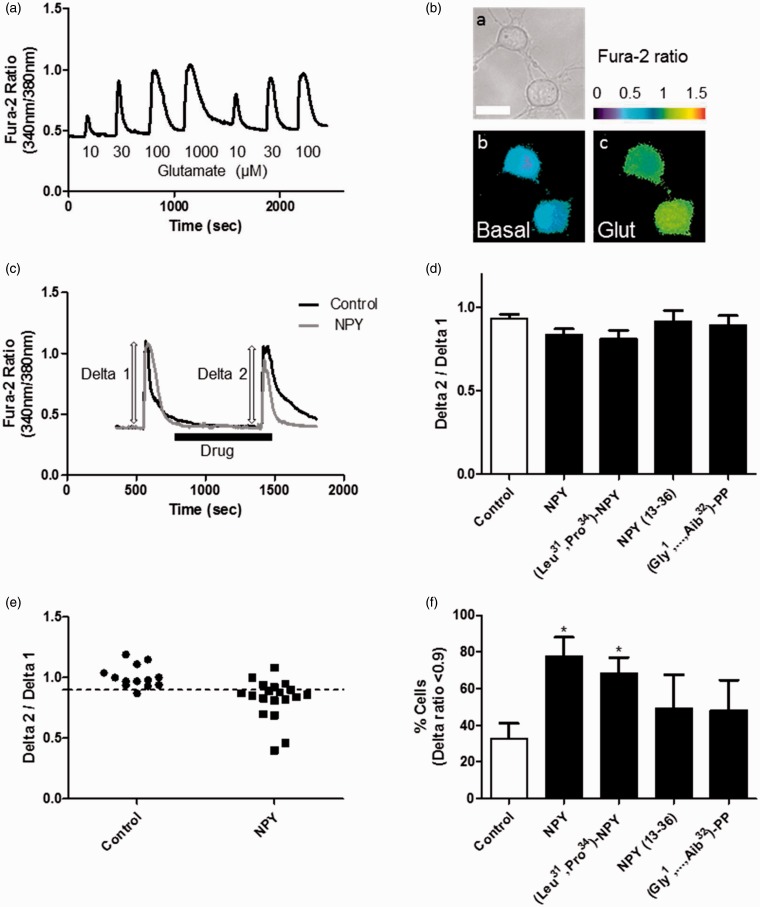
NPY attenuates glutamate-induced [Ca^2+^]_i_ increase in purified RGCs. (a) [Ca^2+^]_i_ trace from a cultured RGC illustrating the response of RGCs to increasing concentrations of glutamate (10–1000 µM). (b) Example of cultured RGCs. Bright field image (ba) and pseudocolor representation of Fura-2 ratio on basal condition (bb) and after stimulation with 30 µM glutamate (bc) are shown. (c) [Ca^2+^]_i_ traces showing RGC responses to two consecutive glutamate stimuli (30 µM) for 30 s each stimulus. After the first glutamate stimulus, 1 µM NPY or a drug-free solution (control) was applied to RGCs during 10 min followed by a second glutamate stimulus. For illustration, two experiments (control and NPY) are shown. The increase above basal Fura-2 ratio (340 nm/380 nm) was quantified for each stimulus as a Delta value. (d) The Delta 2 (second stimulus)/Delta 1 (first stimulus) ratios are presented for different drug applications for 10 min: 1 µM NPY, Figure 3. Continued. 1 µM (Leu^31^, Pro^34^) − NPY, 300 nM NPY_13–36_, 1 µM (Gly^1^, . . . Aib^32^)-PP, or a drug-free solution (control). A small decrease in Delta 2/Delta 1 ratios may be apparent in RGCs exposed to NPY and (Leu^31^, Pro^34^) − NPY. (e) Scatter plots for two populations of RGCs from the same cell culture showing the dispersion of Delta 2/Delta 1 ratio values among cells. Each point represents an individual RGC. The effect of NPY is evident in some RGCs. Cells were treated with a drug-free solution (control) or NPY. Note that in NPY-treated population, a small downward shift may be observed. Dashed line indicates 0.9 ratio value. (f) Percentage of cells presenting Delta 2/Delta 1 ratio below 0.9. The application of NPY or (Leu^31^, Pro^34^) − NPY for 10 min increased the percentage of cells with Delta 2/Delta 1 ratio below 0.9. Data are presented as mean ± SEM of *n* = 5 to 10 independent experiments. *p < .05, compared with control. Kruskal-Wallis followed by Dunn’s test.

We aimed to evaluate the potential modulatory effect of NPY directly on RGCs. For this purpose, we cultured purified RGCs obtained from retinas of P3 to P4 rat pups and performed intracellular free calcium concentration ([Ca^2+^]_i_) measurements using Fura-2 calcium indicator. As previously described (Hartwick et al., 2004), we stimulated RGCs with increasing glutamate concentrations (from 1 to 1000 µM) for 30 s, including also 10 µM glycine (Figure 3(a)). After testing these glutamate concentrations, we used 30 µM glutamate in the following experiments since this concentration induced a submaximal increase in [Ca^2+^]_i_ that was rapidly reversible. The ratios between emission of Fura-2 when excited by light at 340 nm and 380 nm wavelengths were quantified in cell bodies of RGCs as indicative of [Ca^2+^]_i_ (Figure 3(b)). In calibration experiments, we converted Fura-2 ratio to [Ca^2+^]_i_ (see methods section). We obtained basal values of 54 ± 6 nM, while after stimulation with 30 µM glutamate for 30 s, a peak [Ca^2+^]_i_ of 733 ± 59 nM was found (data not shown). After the first glutamate stimulus, different drugs were applied for 10 min: NPY (1 µM, n = 6 independent RGC cultures), the Y_1_/Y_5_ agonist (Leu^31^, Pro^34^)−NPY (1 µM, *n* = 8), the Y_2_ agonist NPY_13–36_ (300 nM, *n* = 5), and the Y_5_ agonist (Gly^1^,…Aib^32^)-PP (1 µM, *n* = 5), or a drug-free solution (control, *n* = 10), followed by a second glutamate stimulus coapplied with the drug (Figure 3(c)). The increase above basal Fura-2 ratio was quantified for each stimulus as a Delta value. When evaluating the average [Ca^2+^]_i_ responses of the cell population analyzed, a small decrease in Delta 2/Delta 1 ratios was found for NPY (0.83 ± 0.04) and (Leu^31^, Pro^34^)−NPY (0.81 ± 0.05), compared with control (0.93 ± 0.02) (Figure 3(d)). In fact, a small significant change may be hard to detect in the overall population, as can be observed in scatter plots showing individual RGCs from an independent experiment (Figure 3(e)). Hence, we quantified the percentage of RGCs where the Delta 2/Delta 1 ratio was lower than 0.9 (Figure 3(f)). The application of NPY or (Leu^31^, Pro^34^)−NPY for 10 min significantly increased the percentage of cells with Delta 2/Delta 1 ratio below 0.9 (77.7 ± 10.3% or 68.5 ± 8.3%, respectively) compared with control (32.7 ± 8.4%). Since the Y_5_ receptor agonist alone was not able to affect the RGC response to glutamate, it is likely that the effect of NPY or (Leu^31^, Pro^34^) − NPY might occur via Y_1_ receptor activation.

### RGC Spiking Activity Modulation via Y_1_ Receptor Activation

**Figure 4.**
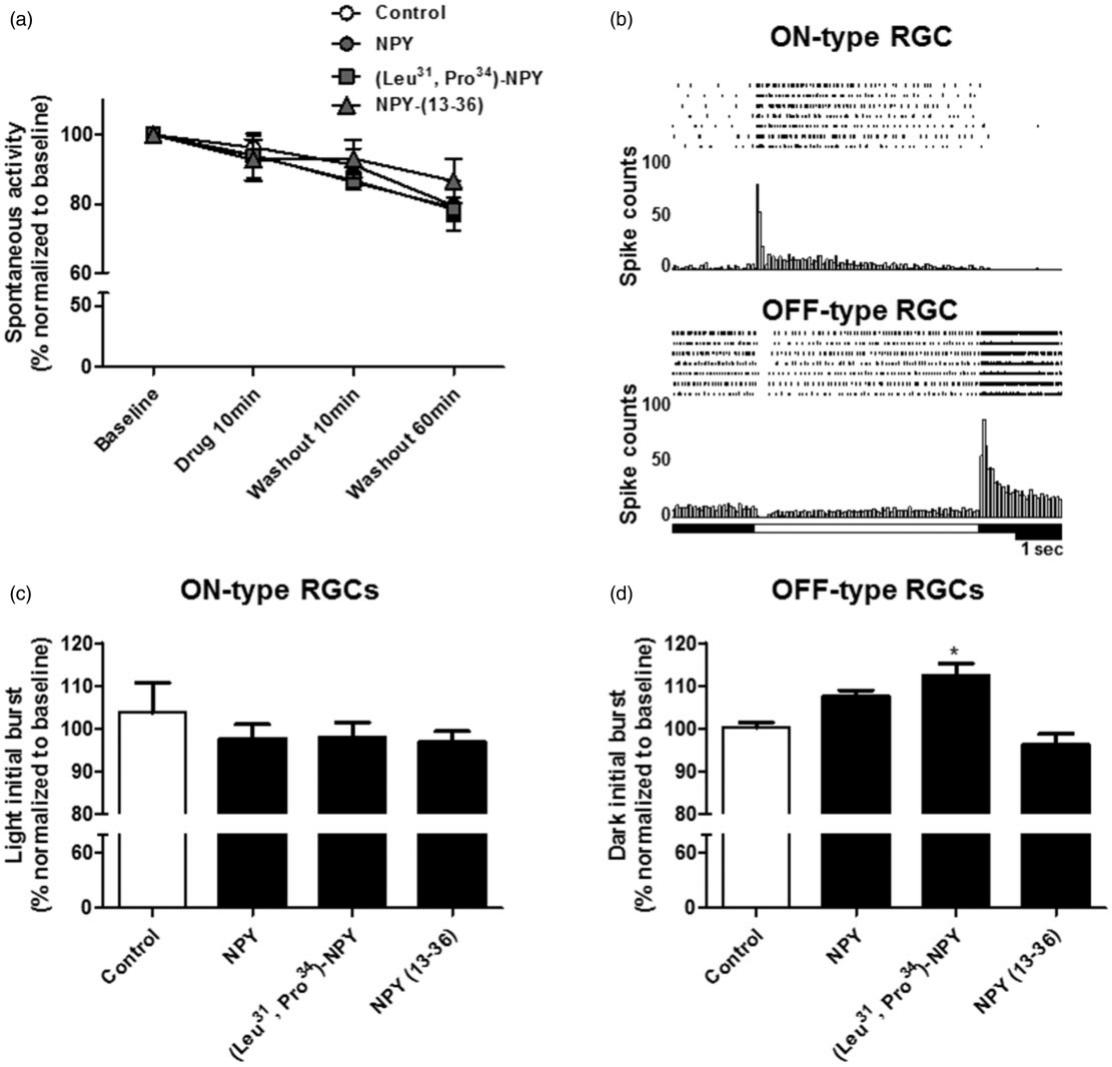
The Y_1_ and Y_5_ receptor agonist (Leu^31^, Pro^34^) − NPY increases the response of OFF-type RGCs to light offset. (a) RGC spontaneous spiking rate quantification after the application of 1 µM NPY, 1 µM (Leu^31^, Pro^34^) − NPY, 1 µM NPY_13–36_, or a drug-free solution (control) for 10 min and upon 60 min of washout was recorded in *ex vivo* retinas using a MEA. A decrease in RGC spiking rate was observed over time, though no effects were found for drug treatments. (b) Examples of peri-stimulus time histograms and raster plots for ON-type RGC and OFF-type RGC responses are shown for seven consecutive stimulus blocks. White rectangles indicate duration of light period. (c) Quantification of initial burst to light onset of ON-type RGCs after application of the same drug treatments as in (a). No effect was found for the different drug treatments compared with control. (d) Quantification of initial burst to light offset of OFF-type RGCs after application of the same drug treatments as in (a). The application of 1 µM (Leu^31^, Pro^34^) − NPY for 10 min was able to increase the magnitude of OFF-type response compared with control. All data were normalized to the values obtained before drug application (baseline). Data are presented as mean ± SEM of *n* = 3 to 4 independent experiments. *p < .05, compared with control. Kruskal-Wallis followed by Dunn’s test.

Since NPY is able to modulate neuronal activity in various brain regions (Silva et al., 2002; Benarroch, 2009), we hypothesized whether application of NPY to *ex vivo* retinas could directly modulate RGC spiking activity. For this purpose, we used a MEA system, which allows simultaneous recording spiking activity from various RGCs. After spike sorting based on template matching, the spiking rate for individual RGCs was quantified. In the first set of experiments, we recorded RGC spontaneous activity (no stimulus) before (baseline) and after the application of NPY (1 µM, *n* = 4 retinas), the Y_1_/Y_5_ agonist (Leu^31^, Pro^34^) − NPY (1 µM, n = 3), the Y_2_ agonist NPY_13–36_ (1 µM, n = 3), or a drug-free solution (control, *n* = 3) for 10 min under continuous perfusion (Figure 4(a)). In addition, a period of washout up to 60 min was included. We found a small decrease in RGC spiking rate over time reaching 79.5 ± 7.2% of baseline in control at 60 min of washout. However, exposure to NPY or NPY receptor agonists caused no effect since RGCs presented a decrease in spiking rate similar to control.

In order to evaluate the effect of NPY on RGC light responses, *ex vivo* retinas were exposed to light stimuli in the photopic range (5.0 cd/m^2^), while RGC spiking activity was recorded with the MEA system. The light stimulation protocol consisted of 10 consecutive stimulation blocks composed of 5 s light followed by 10 s dark. RGC light responses were classified as ON- or OFF-type. An ON-type RGC light response was considered if increased spiking activity was detected at light onset, and an OFF-type RGC light response was considered when increased spiking activity was detected at light offset in each stimulation block. In Figure 4(b), examples of ON- and OFF-type responses are presented. The peri-stimulus time histograms were generated from RGC responses to nine consecutive stimulation blocks and used to calculate the initial burst response to light onset or offset, as well as to estimate the latency of RGC light response. The first RGC light response to a series of 10 stimulation blocks was always omitted from the quantification since it was harder to discriminate it due to the robust spike distortion within burst discharge during light presentation. Thus, in order to evaluate the potential modulatory effect of NPY on RGC light responses, the recordings were performed before (baseline) and after the application of NPY (1 µM, *n* = 4 retinas), the Y_1_/Y_5_ agonist (Leu^31^, Pro^34^) − NPY (1 µM, n = 4), the Y_2_ agonist NPY_13–36_, (1 µM, *n* = 4), or a drug-free solution (control, *n* = 3) for 10 min under continuous perfusion (Figure 4(c) and (d)). Regarding ON-type light response, no apparent effect was found on the initial burst of spiking activity to light onset for the listed above drugs (Figure 4(c)). On the contrary, the application of 1 µM (Leu^31^, Pro^34^) − NPY for 10 min was able to induce a small, but statistically significant, increase to 112.4 ± 2.8% of baseline on the initial burst of spiking activity triggered by ceasing light in OFF-type RGCs (Figure 4(d)), when compared with control (100.3 ± 1.1% of baseline), while no statistically significant effect was found after the application of 1 µM NPY or 1 µM NPY_13–36_. This result suggests that Y_1_ or Y_5_ activation may be sufficient to modulate the OFF-type RGC light response directly by changing the receptive field properties or acting upstream of RGCs in the vertical pathway of visual information, namely at the level of glutamatergic bipolar or photoreceptor cells. When considering the latency of RGC light responses, no effect was found in both ON- or OFF-type RGC responses after the different drug treatments used (data not shown).

**Figure 5.**
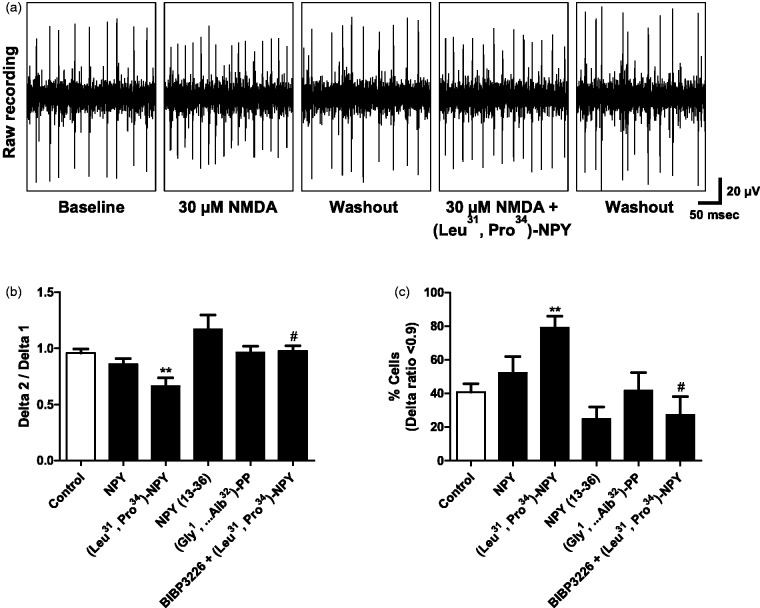
Y_1_ receptor activation decreases NMDA-stimulated RGC spiking activity. (a) Representative recordings of RGC spiking activity in *ex vivo* retina using MEA. Note the increase in spiking rate upon 30 µM NMDA. After washout of first NMDA stimulus, 1 µM (Leu^31^, Pro^34^) − NPY was applied for 10 min followed by a second NMDA stimulus. (b) The Delta 2 (second stimulus)/Delta 1 (first stimulus) ratios are presented for different drug applications, for 10 min, between NMDA stimuli: 1 µM NPY, 1 µM (Leu^31^, Pro^34^) − NPY, 1 µM NPY_13–36_, 1 µM (Gly^1^, . . . Aib^32^)-PP, or a drug-free solution (control). The application of (Leu^31^, Pro^34^) − NPY was able to reduce the NMDA-stimulated RGC spiking activity. This effect was blocked by the Y_1_ receptor antagonist BIBP 3226 (1 µM). (c) Percentage of RGCs presenting Delta 2/Delta 1 ratio below 0.9. The application of (Leu^31^, Pro^34^) − NPY significantly increased the percentage of cells with Delta 2/Delta 1 ratio below 0.9. BIBP 3226 was able to block the effect of (Leu^31^, Pro^34^) − NPY confirming the involvement of Y_1_ receptor activation. Data are presented as mean ± SEM of *n* = 3 to 10 independent experiments. **p < .01, compared with control; ^#^*p* < .05, compared with (Leu^31^, Pro^34^) − NPY. Kruskal-Wallis followed by Dunn’s test.

We also evaluated the potential modulatory effect of NPY application on RGC spiking activity upon glutamate receptor activation. In this experimental set, no light stimulation was applied. Glutamate is easily cleared within the retina by glutamate uptake (Higgs and Lukasiewicz, 1999), and therefore, we applied NMDA directly to *ex vivo* retinas in order to induce synaptic excitation of RGCs, since NMDA receptors are abundantly expressed in RGCs (Shen et al., 2006). The application of NMDA (30 µM) for 5 min induced an acute increase in RGC spiking activity, which was reversed by 10 min of washout (Figure 5(a)). After washout of the first NMDA stimulus, different drugs were applied for 10 min under continuous perfusion: NPY (1 µM, *n* = 8 retinas), the Y_1_/Y_5_ agonist (Leu^31^, Pro^34^) − NPY (1 µM, *n* = 8), the Y_2_ agonist NPY_13–36_ (1 µM, *n* = 6), the Y_5_ agonist (Gly^1^,…Aib^32^)-PP (1 µM, *n* = 5), or a drug-free solution (control, *n* = 10). After 10 min of drug treatment, a second NMDA stimulus was coapplied, followed by 10 min of washout. The Delta value represents the difference between the maximum spiking frequency (or spiking rate) during NMDA application and the spiking frequency obtained before NMDA exposure. Subsequently, the Delta 2 (second stimulus)/Delta 1 (first stimulus) ratios were calculated for each individual RGC. We found that application of (Leu^31^, Pro^34^) − NPY was able to decrease the Delta 2/Delta 1 ratio to 0.67 ± 0.07, when compared with control, 0.96 ± 0.04 (Figure 5(b)), indicating that activation of Y_1_ or Y_5_ receptor was sufficient to attenuate the NMDA-stimulated RGC spiking activity. For the other drug treatments, no statistically significant alterations were detected compared with control. The effect of (Leu^31^, Pro^34^) − NPY was demonstrated to be mediated by Y_1_ receptor activation since the coapplication of Y_1_ receptor antagonist BIBP 3226 (1 µM, n = 3) and (Leu^31^, Pro^34^) − NPY was able to prevent the agonist effect (Figure 5(b)). Moreover, the application of the Y_5_ agonist alone did not affect the RGC response to NMDA, suggesting that the effect found for (Leu^31^, Pro^34^) − NPY is not mediated by Y_5_ receptor activation. Focusing on the RGCs that presented more pronounced differences between responses to first and second NMDA stimulus, we calculated the percentage of RGCs presenting Delta 2/Delta 1 ratio values below 0.9 for each independent experiment (Figure 5(c)), thus, overcoming the masking effect associated with overall population mean calculation. As expected, the application of (Leu^31^, Pro^34^) − NPY increased the percentage of cells (79.0 ± 6.9% of the cells analyzed) with Delta 2/Delta 1 ratio below 0.9, when compared with control (40.8 ± 5.0% of the cells analyzed; Figure 5(c)). Also, the Y_1_ receptor antagonist, BIBP 3226, was able to prevent the effect of (Leu^31^, Pro^34^) − NPY, confirming the involvement of Y_1_ receptor activation. No statistically significant difference was detected for the other drugs. We hypothesized that a possible opposite effect of Y_2_ activation might be responsible for counteracting the effect of Y_1_ activation by NPY. In this regard, the application of Y_2_ receptor agonist NPY_13–36_ resulted in a Delta 2/Delta 1 ratio of 1.2 ± 0.1 (Figure 5(b)), and the percentage of cells with Delta 2/Delta 1 ratio below 0.9 was 24.8 ± 7.2% (Figure 5(c)), both values apparently in opposite direction of Y_1_ receptor activation, although no statistically significant difference was detected.

### NPY Reduces NMDA-Induced Cell Death in the GCL in Retinal Explants via Y_1_ and Y_5_ Receptor Activation

**Figure 6.**
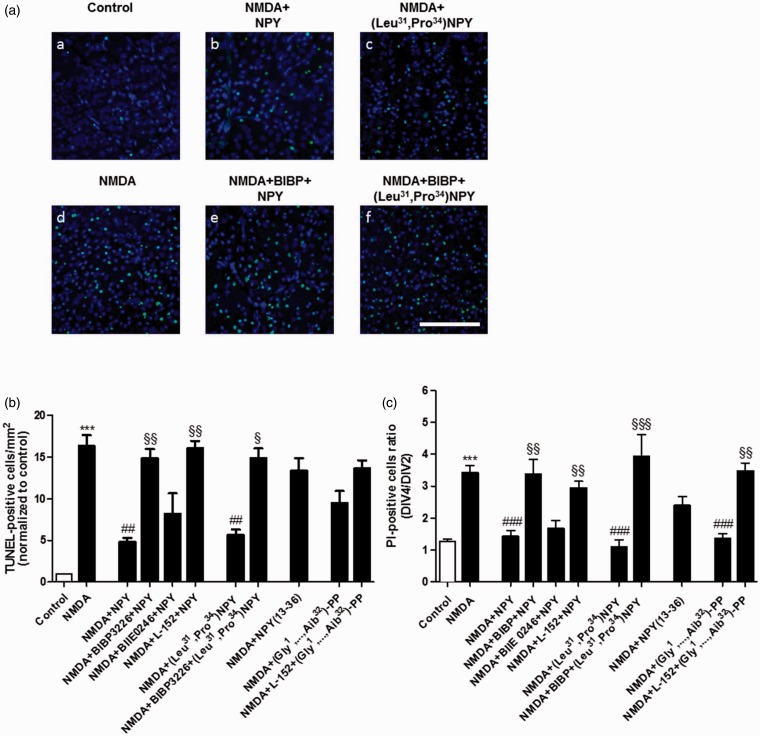
NPY reduces NMDA-induced cell death in the GCL of retinal explants via Y_1_ and Y_5_ receptor activation. Retinal explants were exposed to 300 µM NMDA for 48 h to induce an excitotoxic insult. The agonists, 1 µM NPY, 1 µM (Leu^31^, Pro^34^) − NPY, 300 nM NPY_13–36_, and 1 µM (Gly^1^, . . . Aib^32^)-PP, were applied 24 h and 60 min before NMDA application. All the antagonists: 1 µM BIBP 3226, 1 µM BIIE 0246, and 1 µM L-152,804 were applied 30 min before the agonist. (a) Representative images of TUNEL-positive cells (green) in the GCL of control (aa) or NMDA-treated (ab-af) retinal explants. Nuclei were stained with DAPI (blue). Retinal explants were pretreated with different drugs 24 h before exposure to NMDA. Scale bar: 100 µm. (b) Quantification of TUNEL-positive cells. NMDA exposure induced a significant increase in the number of TUNEL-positive cells in the GCL compared with control. Pretreatment with 1 µM NPY or 1 µM (Leu^31^, Pro^34^) − NPY (Y_1_/Y_5_ receptor agonist), 24 h and 60 min before NMDA, was able to reduce the increase in the number of TUNEL-positive cells induced by NMDA. Pretreatment with Y_1_ or Y_5_ receptor antagonist (1 µM BIBP 3226 or 1 µM L-152,804, respectively), when applied 30 min before NPY, blocked its protective effect. The effect of (Leu^31^, Pro^34^) − NPY was blocked by BIBP 3226. (c) NMDA exposure induced a significant increase in the number of PI-positive cells in retinal explants. Data are presented as the ratio between PI-positive cells at DIV4 and DIV2. NPY or (Leu^31^, Pro^34^) − NPY was able to reduce the increase in the number of PI-positive cells induced by NMDA. BIBP 3226 or L-152,804 blocked the protective effect of NPY, and BIBP 3226 blocked the protective effect of (Leu^31^, Pro^34^) − NPY. The application of 1 µM (Gly^1^, . . . Aib^32^)-PP was able to reduce the increase in PI-positive cells induced by NMDA, and this effect was blocked by L-152,804. Together, the results from TUNEL and PI assays suggest that NPY is able to protect retinal cells from an excitotoxic insult through the activation of Y_1_ and Y_5_ receptors. Data are presented as mean ± SEM of *n* = 5 to 11 independent experiments. ***p < .001, compared with control; ^##^*p* < .01, ^###^*p* < .001, compared with NMDA; ^§^*p* < .05, ^§§^*p* < .01, ^§§§^*p* < .001, compared with NMDA + NPY, or NMDA + (Leu^31^, Pro^34^) − NPY, or NMDA + (Gly^1^, . . . Aib^32^)-PP. Kruskal-Wallis followed by Dunn’s test.

NPY exerts neuroprotective actions in various brain regions, and also in the retina (Alvaro et al., 2008b; Santos-Carvalho et al., 2013a), suggesting that NPY receptors might be possible therapeutic targets for retinal degenerative diseases such as glaucoma. Therefore, in addition to the NPY modulatory effect on RGC activity detected in *ex vivo* retinas, we also evaluated whether NPY could be able to prevent retinal cell death induced by an excitotoxic insult. The excitotoxic insult was induced in retinal explants (cultured for four DIV) exposed to 300 µM NMDA for 48 h. Apoptotic retinal cell death was assessed by TUNEL assay, and necrotic or late apoptotic cell death was assessed by PI incorporation assay (Figure 6). Different drug treatments were applied to assess the potential protective effect of NPY receptor activation. Thus, NPY (1 µM, *n* = 6 retinas for TUNEL, *n* = 9 retinas for PI), the Y_1_/Y_5_ agonist (Leu^31^, Pro^34^) − NPY (1 µM, *n* = 6 for TUNEL, *n* = 7 for PI), the Y_2_ agonist NPY_13–36_, (300 nM, *n* = 6 for TUNEL, *n* = 11 for PI), the Y_5_ agonist (Gly^1^,…Aib^32^)-PP (1 µM, *n* = 6 for TUNEL, *n* = 6 for PI), or a drug-free solution (control, *n* = 11 for TUNEL, *n* = 10 for PI), were applied at DIV1 and DIV2, respectively 24 h and 60 min before NMDA treatment.

Exposure of retinal explants to NMDA for 48 h increased the number of TUNEL-positive cells in the GCL, up to 16.3 ± 1.3 times higher than in control (Figure 6(b)). There was also an increase in the DIV4/DIV2 ratio for PI-positive cells compared with control, namely 3.4 ± 0.2 in NMDA treated explants versus 1.3 ± 0.1 in control (Figure 6(c)). Pretreatment with NPY was able to reduce the increase in the number of TUNEL-positive cells induced by NMDA (4.8 ± 0.5 times higher than in control). NPY pretreatment was also able to reduce the increase in DIV4/DIV2 ratio for PI-positive cells (1.4 ± 0.2; Figure 6(c)). The Y_1_ antagonist BIBP 3226 (1 µM, *n* = 5 for TUNEL, *n* = 7 for PI) or the Y_5_ antagonist L-152,804 (1 µM, *n* = 5 for TUNEL, *n* = 9 for PI), when applied 30 min before NPY, was able to block its protective effect both on TUNEL and PI assays, indicating the involvement of Y_1_ and Y_5_ receptors on the neuroprotective effects of NPY. The protective effect of Y_1_ receptor activation was confirmed also by the application of (Leu^31^, Pro^34^) − NPY, which was able to reduce the NMDA-induced increase in TUNEL- and PI-positive cells. Again, the Y_1_ antagonist BIBP 3226 (*n* = 6 for TUNEL, *n* = 5 for PI) blocked this effect. Concerning the Y_5_ receptor activation, the use of (Gly^1^, . . . Aib^32^)-PP reduced the number of TUNEL-positive cells comparing to NMDA-treated explants, though no statistically significant difference was detected. Accordingly, Y_5_ receptor activation was able to reduce the number of PI-positive cells compared with NMDA condition. This later effect was blocked by L-152,804 (*n* = 5 for TUNEL, *n* = 5 for PI), thus confirming the protective effect of Y_5_ receptor activation. The Y_2_ receptor was not involved in the protective action of NPY since the Y_2_ antagonist BIIE 0246 (1 µM, n = 5 for TUNEL, n = 8 for PI) did not block the neuroprotective effect of NPY. Also, NPY_13–36_ did not reduce the increase in TUNEL- and PI-positive cells induced by NMDA in retinal explants.

### Intravitreal Administration of NPY or (Leu^31^, Pro^34^) − NPY Does Not Prevent Retinal Cell Death Induced by I-R Injury

**Figure 7.**
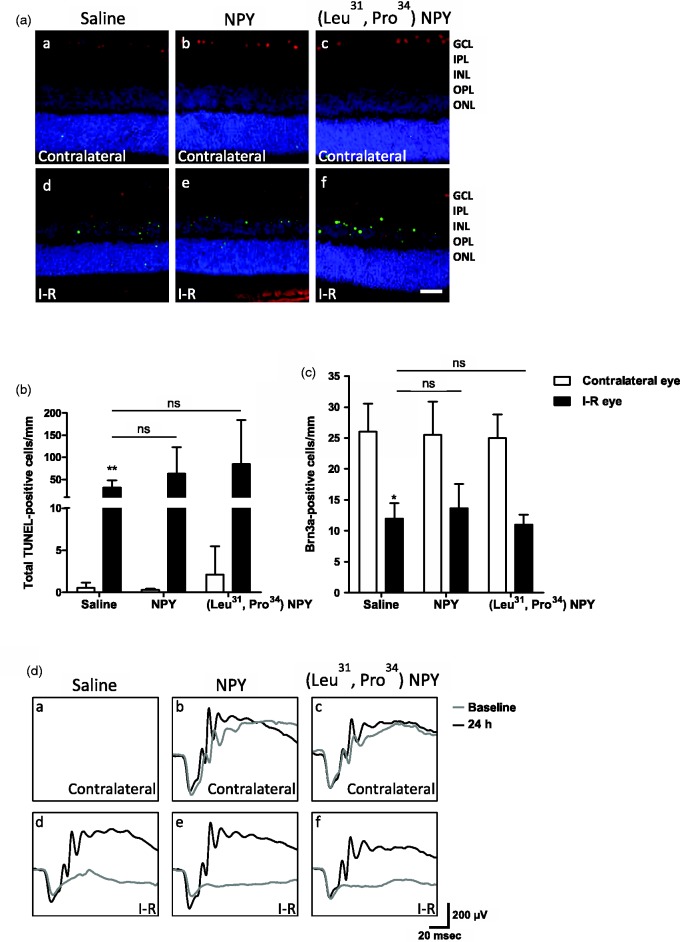
Intravitreal administration of NPY or (Leu^31^, Pro^34^) − NPY did not prevent cell death induced by retinal I-R injury at 24 h of reperfusion. Retinal ischemia was induced for 60 min followed by 24 h of reperfusion. Ischemia was induced in one eye, and the contralateral eye was taken as an internal control. (a) Representative images of retinal sections showing TUNEL-positive cells (green) and Brn3a-positive RGCs (red). Nuclei were stained with DAPI (blue). Saline (aa, ad), 10 µg (2.34 nmol) NPY (ab, ae), or 10 µg (2.36 nmol; Leu^31^, Pro^34^) − NPY (ac, af) were intravitreally injected 2 h before the onset of I-R injury. GCL = ganglion cell layer; IPL = inner plexiform layer; INL = inner nuclear layer; OPL = outer plexiform layer; ONL = outer nuclear layer. Scale bar: 50 µm. (b) TUNEL-positive cells are Figure 7. Continued. expressed per mm of section length. I-R injury induced an increase in the number of TUNEL-positive cells. NPY or (Leu^31^, Pro^34^) − NPY administration was not able to reduce the number of TUNEL-positive cells. (c) Brn3a-positive RGCs were expressed per mm of section length. NPY or (Leu^31^, Pro^34^) − NPY administration was not able to prevent the reduction in Brn3a-positive RGC number induced by I-R injury. (d) Examples of scotopic ERG traces for saline (da, dd), NPY-treated (db, de), or (Leu^31^, Pro^34^) − NPY-treated eyes (dc, df). ERG recordings were performed before (baseline) and 24 h after I-R injury. Note that in the injured eye the b-wave was reduced (dd). NPY or (Leu^31^, Pro^34^) − NPY administration did not prevent the reduction in b-wave (de, df). Data are presented as mean ± SEM of *n* = 5 to 6 independent experiments. *p < .05, **p < .01, compared with contralateral eye. Kruskal-Wallis followed by Dunn’s test.

In a previous study from our group, pretreatment with NPY before the intravitreal injection of glutamate was shown to afford neuroprotection to RGCs (Santos-Carvalho et al., 2013a). Here, we explored the potential neuroprotective action of NPY in an animal model of retinal damage induced by I-R injury (Figure 7). Saline (*n* = 5 animals), 10 µg (2.34 nmol) NPY (*n* = 6), or 10 µg (2.36 nmol; Leu^31^, Pro^34^) − NPY (Y_1_/Y_5_ receptor agonist, *n* = 5) were intravitreally injected 2 h prior to I-R injury. Retinal cell death was assessed by TUNEL assay. I-R injury induced an increase in the number of TUNEL-positive cells (31.8 ± 6.6) per mm of retinal section length across all the retinal nuclear layers compared with the retina of the contralateral eye (0.6 ± 0.3 TUNEL-positive cells; Figure 7(b)). This increase was not prevented by pretreatment with NPY or (Leu^31^, Pro^34^) − NPY. RGC survival was evaluated by counting the number of Brn3a-immunoreactive cells, a RGC marker Brn3a (Nadal-Nicolas et al., 2012). We found that I-R injury decreased the number of Brn3a-positive RGCs per mm of retinal section length to 12.0 ± 2.5 RGCs compared with the contralateral eye (26.0 ± 4.5 Brn3a-positive cells), and again, this effect was not prevented by NPY or (Leu^31^, Pro^34^) − NPY administration (Figure 7(c)). We also performed ERGs in order to assess the functional effect of I-R injury on retinal light responses and a potential protective effect of NPY. Scotopic and photopic ERGs were recorded before the onset of I-R injury (baseline) and after 24 h of reperfusion (Figure 7(d)). We found that I-R injury induced a clear decrease in scotopic and photopic b-wave amplitudes but not in scotopic a-wave amplitude (data not shown). Similarly to results on TUNEL and Brn3a assays, the NPY or (Leu^31^, Pro^34^) − NPY administration before I-R injury did not prevent the reduction in ERG b-wave amplitudes. Together, these results indicate that, contrarily to the protective action against an excitotoxic injury in retinal explants, NPY or (Leu^31^, Pro^34^) − NPY pretreatment was not able to prevent retinal I-R injury within 24 h of reperfusion.

## Discussion

**Figure 8.**
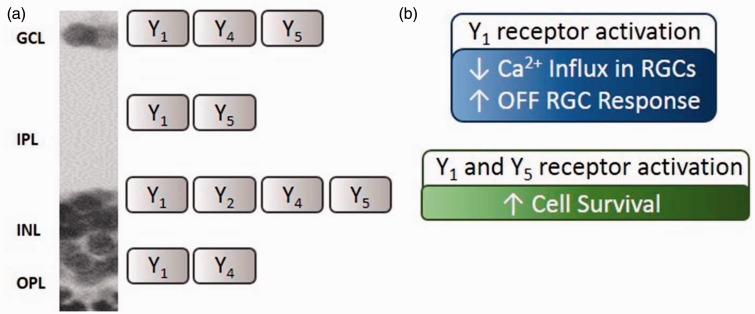
NPY receptor activation leads to RGC modulation and increases cell survival in the inner retina. (a) NPY receptor location in the different layers of the adult rat retina. (b) The activation of Y_1_ receptor leads to decreased Ca^2+^ influx in RGCs, and also increased OFF RGC response, potentially by inhibiting ON-driven surround antagonism in OFF-center RGCs. The activation of Y_1_ in RGCs and amacrine cells and Y_5_ in Müller cells leads to increased cell survival in the inner retina. GCL = ganglion cell layer; IPL = inner plexiform layer; INL = inner nuclear layer; OPL = outer plexiform layer.

The presence of mRNA encoding NPY and NPY receptors (Y_1_, Y_2_, Y_4_, and Y_5_) in the rat retina has been demonstrated in previous studies (D’Angelo and Brecha, 2004; Alvaro et al., 2007). We here present evidence that NPY and NPY receptors are specifically present in freshly isolated RGCs (Figure 1). NPY-ir in the retina has been evaluated in different species (Santos-Carvalho et al., 2014). In human retina, NPY-ir was found in a subset of amacrine cells and RGCs, with processes extending mainly in the IPL (Tornqvist and Ehinger, 1988; Straznicky and Hiscock, 1989). In rat retina, NPY-ir was reported to localize to cell bodies of amacrine cells in INL and displaced amacrine cells in GCL, and to colocalize mainly with GABAergic neurons. The corresponding cell processes extend and ramify mainly in strata 1, 3, and 5 of IPL (Oh et al., 2002). We confirmed these observations using retinal slices of adult rats (Figure 1). In particular, we found NPY-ir in a purified culture of RGCs obtained from P3 to P4 pups. Previously, we also suggested the presence of NPY-ir in different retinal cell types in primary cultures (Alvaro et al., 2007). Regarding NPY receptor localization, only a few studies addressed this issue. Y_1_ receptor-ir was detected in glial cells of diseased human retina and in horizontal and amacrine cells of rat retina (Canto Soler et al., 2002; D’Angelo et al., 2002), and we have previously detected immunoreactivity for Y_1_ and Y_2_ receptors in neurons and glial cells in cultured retinal cells (Santos-Carvalho et al., 2013b). Moreover, we and other authors have provided functional evidence for the presence of Y_1_, Y_2_, Y_4_, and Y_5_ receptors in retinal cells (Bruun et al., 1994; D’Angelo and Brecha, 2004; Milenkovic et al., 2004; Alvaro et al., 2008a; Alvaro et al., 2009; Santos-Carvalho et al., 2013a). Regarding purified RGC cultures, we detected immunoreactivity for NPY and Y_1_, Y_2_, Y_4_, and Y_5_ receptors in Brn3a-positive RGCs. In retinal sections, Y_1_-ir was found in INL and strata 2 and 4 of IPL as previously reported (D’Angelo et al., 2002). In addition, we detected Y_1_-ir RGCs (Figure 8), which is in agreement with the labeling found in purified RGC cultures. We described, for the first time, the localization of Y_2_ receptor-ir in stratum 1 of IPL and in cell bodies in proximal INL. We also detected for the first time Y_4_ and Y_5_ receptor-ir in retinal sections. Y_4_ receptor-ir was localized to cell bodies of GCL, and proximal and distal INL. The cell bodies in GCL that were immunoreactive for Y_4_ receptor were both RGC (colocalized with Brn3a) and nonBrn3a-positive cells, likely displaced amacrine cells. Concerning the Y_5_ receptor, immunoreactivity was detected in Müller cells. The lack of clear immunoreactivity for NPY, and for Y_2_ and Y_5_ receptors in RGCs in retinal sections of adult rat, while it could be found in cultured RGCs from P3 to P4 rats, may be explained by decreased expression in adulthood or related with cell culture conditions, which might favor the expression of NPY and NPY receptors. We also found functional active NPY receptors in the inner retina using [^35^S]GTPγS-binding assay (Figure 2). Binding signal detected in the photoreceptor layer might represent the high amount of G proteins in photoreceptor outer segments, mainly transducin (Arshavsky et al., 2002).

NPY has been associated with inhibitory actions after electrical or chemical stimulation of excitatory synaptic transmission (El Bahh et al., 2002; Tu et al., 2006; Kovac et al., 2011). In fact, NPY was reported to decrease depolarization-evoked increase in [Ca^2+^]_i_ by inhibiting voltage-dependent Ca^2+^ channels. This effect was reported in a variety of neuronal cell types such as dorsal root ganglion neurons (Bleakman et al., 1991), hippocampal neurons (Bleakman et al., 1992), submandibular ganglion neurons (Endoh et al., 2012), and hypothalamic arcuate nucleus neurons (Sun and Miller, 1999). Moreover, the activation of Y_1_ receptors has been associated with inhibition of neurotransmitter release (Bitran et al., 1999; Hastings et al., 2004; Wang, 2005), though the opposite behavior can be found depending on the neurotransmitter or cell type (Hastings et al., 2001). In the retina, Y_2_ receptor activation attenuated depolarization-evoked Ca^2+^ influx in rod bipolar cell terminals (D’Angelo and Brecha, 2004). In addition, previous work in our laboratory has shown that NPY application to retinal cell cultures was able to reduce depolarization-evoked [Ca^2+^]_i_ increase via activation of Y_1_ and Y_5_ receptors (Alvaro et al., 2009). Thus, the present study was aimed at evaluating the effect of direct application of NPY or NPY receptor agonists on [Ca^2+^]_i_ changes in RGCs, using a purified RGC culture.

Glutamate is the major retinal excitatory neurotransmitter mediating the transmission of visual information from bipolar cells to RGCs. In this experiment, 30 µM glutamate plus 10 µM glycine was used to stimulate Ca^2+^ influx into cultured RGCs by activating both NMDA and nonNMDA glutamate receptors and indirectly voltage-dependent calcium channels, as previously described (Hartwick et al., 2004). However, under these conditions, Ca^2+^ influx was found to be primarily mediated by NMDA glutamate receptors (Hartwick et al., 2008). We now present new evidence showing an inhibitory effect of NPY on the glutamate-evoked increase in [Ca^2+^]_i_ in purified RGCs through Y_1_ receptor activation (Figure 3). This result suggests that NPY, released from amacrine cells in the rat retina (Oh et al., 2002), may activate postsynaptic Y_1_ receptors on RGCs inhibiting glutamate receptors or voltage-dependent calcium channels, thus modulating the effect of glutamate released from bipolar cells (Figure 8). The specific activation of Y_2_ or Y_5_ receptors did not evoke any significant change in [Ca^2+^]_i_ in purified RGCs (Figure 3(f)), although these receptors were expressed in similar purified RGC cultures (Figure 1(b)). This may result from the presence of nonfunctional Y_2_ and Y_5_ receptors in these cultures.

We further explored the modulatory potential of NPY on RGC spiking activity using a MEA technique (Meister et al., 1994). Although application of NPY or NPY receptor agonists tested did not affect the spontaneous spiking activity, a small but statistically significant increase was found at the initial burst response of OFF-type RGCs upon the application of Y_1_ and Y_5_ receptor agonist (Leu^31^, Pro^34^) − NPY (Figures 4 and 8). We speculate that the modulatory effects of NPY receptor activation may be exerted within the circuitry generating center-surround organization within the receptive field of OFF-type RGCs at the level of amacrine cell-RGCs (Sinclair et al., 2004). A decreased contribution of ON bipolar cell signal to this circuitry might not be responsible for this effect since activation of Y_2_ receptors, but neither Y_1_ nor Y_5_, was reported to inhibit rod bipolar cells (D’Angelo and Brecha, 2004). Another possibility is that the modulatory mechanism may reside at the level of horizontal cell circuitry in OPL, since it contributes to center-surround organization (Wassle and Boycott, 1991), and the Y_1_ receptor localizes to rat horizontal cells (D’Angelo et al., 2002).

In another experimental paradigm, *ex vivo* rat retinal preparations were exposed to NMDA in order to induce an excitation of RGCs. We chose NMDA instead of glutamate since in intact retina even high concentrations of glutamate have no effect on [Ca^2+^]_i_ in RGCs (Hartwick et al., 2004), due to the presence of glutamate uptake systems (Thoreson and Witkovsky, 1999). We clearly show for the first time that Y_1_ receptor activation is able to modulate directly RGC responses by attenuating the NMDA-induced increase in RGC spiking activity (Figure 5). A possible effect of NPY application cannot be excluded, but it might be masked by an effect of Y_2_ activation counteracting the effect of Y_1_ activation by NPY. These results suggest that the activation of Y_1_ receptors present on RGC dendrites is responsible for the modulatory effect observed. Nevertheless, the contribution of Y_1_ receptor expressing amacrine cell inputs onto RGC dendrites may also be important in this modulation (D’Angelo et al., 2002). Additional neuroanatomical and electrophysiological studies are needed to clarify the cellular contribution (RGCs vs. amacrine cells) and localization of Y_1_ receptor activation responsible for the alterations in RGC spiking activity, as well as whether Y_1_ receptor activation is associated with a particular RGC type or modulates a specific visual task (Gollisch and Meister, 2010).

NPY has been shown to prevent neuronal cell death, also in the retina, induced by excitotoxic insults (Silva et al., 2003; Xapelli et al., 2007; Santos-Carvalho et al., 2013a). To further address the potential neuroprotective role of NPY in the retina, we used an *in vitro* model, cultured retinal explants exposed to NMDA, as well as an animal model of retinal I-R injury. Both cultured retinal explants and the I-R model have been previously used to evaluate neuroprotective strategies targeting retinal neurons, specially RGCs (Lagreze et al., 1998; Goodyear and Levin, 2008; Peng et al., 2011; Zhang et al., 2012). We now found that NPY pretreatment was able to prevent NMDA-induced cell death in retinal explants, through the activation of Y_1_ and Y_5_ receptors (Figures 6 and 8). This result is in agreement with previous studies showing NPY neuroprotective effects under activation of Y_1_ or Y_5_ receptors (Silva et al., 2003; Xapelli et al., 2007; Smialowska et al., 2009), including in retinal neurons (Santos-Carvalho et al., 2013a). However, in I-R injury model, pretreatment with NPY or (Leu^31^, Pro^34^) − NPY was not able to prevent cell death or rescue RGCs (Figure 7). This observation contrasts with the protective effect of NPY when injected intravitreally before the intravitreal injection of glutamate, in a model of glutamate-induced injury in the retina (Santos-Carvalho et al., 2013a). In fact, there is a complex involvement of a series of events leading to cell injury triggered by I-R, compared with the exposure to glutamate injected into the vitreous. Those events include primarily the formation of free radicals during the early stage of reperfusion which overwhelms normal cellular antioxidant defense mechanisms, as well as inflammation and excitotoxicity (Osborne et al., 2004). Taking into account the protective effects we found using the glutamate intravitreal injection model as well as the protective effect in retinal explants, clearly indicating that NPY can be neuroprotective in the retina, it is likely that a protective effect could be obtained with a less severe insult. Therefore, further studies are needed in order to evaluate whether NPY neuroprotective effects detected in cultured retinal explants can be translated into animal models of retinal degenerative diseases.

## Summary

In this study, we found that neuropeptide Y (NPY) and NPY receptors are present in retinal ganglion cells (RGCs). The activation of Y_1_ receptor modulates intracellular calcium levels and also spiking activity in RGCs.
